# Observations of the Pathology and Treatment of Cholera Infantum

**Published:** 1832

**Authors:** John Wiltbank

**Affiliations:** Philadelphia


Art. II.—*Observations on the Pathology, and treatment of Cholera Infantum.* By John Wiltbank, M. D., of Philadelphia.

When we take into consideration the character of this disease, its extensive prevalence, and the desolating ravages that annually attend its course in our large cities, we will be led duly to estimate the importance of a correct knowledge of its pathology, upon which we may found a mode of treatment, calculated to lessen its mortality, and to prevent its wide-spreading prevalence. Medical writers have paid but little attention to Cholera Infantum; and it is equally a subject of surprise and regret, that while upon every other malady that afflicts the human system, volume upon volume has been written and largely commented upon, the amount of our information upon this disease, is contained in fugitive essays, and slight notices, scattered through our journals; and in a few general observations to be met with in our regular treatises upon the practice of medicine. This is a stigma upon us, as American Physicians, that I sincerely hope will soon be removed, by the publication of a volume, devoted to this subject, by some one whose experience, observation and industry, may well qualify him to accomplish it. The appearance of such a work would be hailed as a public benefit, and its author rewarded by the meed of his own approbation, and by that of his Medical brethren and the public at large. Though I have not the presumption to suppose, that I can, to any degree, supply this deficiency, yet it gives me pleasure to add my mite of information to the general stock, in the anticipation that the disease will soon receive the attention it merits, from one more competent to do justice to the subject.

Cholera Infantum assumes a variety of forms, as it occurs in different individuals, or is modified by different causes. Diarrhœa, dysentery and cholera morbus, are all imitated by it, and according as it assumes the symptoms of one or other of these diseases, it is likely to prove curable, or perhaps, inevitably fatal.

*Symptoms.* An attack of cholera infantum is sometimes preceded a few days, by nausea, loss of appetite, thirst and a slight diarrhœa. It usually, however, makes its attack more suddenly and with greater violence, by intense thirst, incessant vomiting, griping pains and frequent discharges of unnatural stools from the bowels. The general system soon sympathises and fever ensues. The fever appears to be of an irregular remitting type, with evident exacerbations, especially in the evening.—The pulse is at the onset of the disease weak, small and irritated, becoming more frequent and corded, but never strong or full. The skin is harsh and dry, or it may be cold and clammy and of unequal temperature, the head and abdomen being hot, while the extremities are cold. The stools are usually frequent and unnatural, and rarely large in quantity. Their appearance varies, being, in different cases, white, yellow, green, watery, frothy, mucous, bloody, or scybalous; and they may be extremely offensive or nearly inodorous. In some instances, they resemble the washings of putrid meat both in colour and smell. The appearance of bile, which may, in the early stages, have been quite evident, is now wholly removed. The little sufferer becomes restless and uneasy, and is never quiet in one posture—the bowels are spasmodically affected, and become so irritable, that the food passes through them unchanged, as in lyentery. The brain very early shows its participation in the diseased action—the eyes are either wild and fierce in their expression, or they may be remarkably placid and serene: the temples throb, and delirium and convulsions not unfrequently close the scene.

Such is a brief sketch of the symptoms that usually attend the acute stage of this affection. The disease becoming chronic, however, the symptoms become highly aggravated—The brain, which has hitherto exhibited the signs of irritation, is now much more seriously implicated.

Dr. Rush, in his excellent remarks upon this disease, observes, "that the head is so much affected, as in some instances, to produce symptoms, not only of delirium, but of mania, insomuch that the children throw their heads backwards and forwards, and sometimes make attempts to scratch, and to bite their parents, nurses, and even themselves." "Such," he continues, "is the insensibility of the system in some instances of this disease, that flies have been seen to alight upon the eyes when open, without exciting a motion in the eyelids to remove them." Emaciation now rapidly ensues, and the expression of the countenance is entirely changed. The lips are swollen, the eyes dull, heavy, and sunk, and during sleep half open; the skin is shrivelled and drawn tight over the forehead: the abdomen becomes distended, painful to the touch, and hard, aphthæ appear about the mouth and fauces, and the legs and feet become œdematous. The purging is now incessant, the fæces being discharged without effort, forty or fifty times in the course of the day. So great is the irritability of the alimentary canal, that the most bland and digestible articles of diet are rejected, often before they reach the stomach, or pass through the bowels unaltered. In this state, our little patient will sometimes remain, with little variation, one or two months, and ultimately recover. When this is not the case, the child becomes comatose and insensible, spots of effused blood appear under the cuticle, and the symptoms of hydrocephalus hurry him to the grave.

In forming our *diagnosis* of this disease, we cannot well err. The season of the year in which it occurs—its extensive prevalence in our large cities, and its characteristic symptoms distinguish it from all other diseases. The only diseases to which it bears any affinity are diarrhœa, dysentery, and cholera morbus, and the treatment being so nearly similar in these cases, no nicety of discrimination is required.

*The prognosis* is by no means easy. Children will frequently languish under the most violent forms of the disease six or eight weeks, and ultimately recover, while others will suddenly, and contrary to our most reasonable expectations, fall victims to its violence in a few hours. We may, however, predict with some degree of certainty, a favourable termination, when the pulse becomes more full and slow, the skin soft and of a uniform temperature, the discharges from the bowels less frequent and painful and, at the same time, more copious and of a dark bilious appearance, and the cerebral irritation subsides, as is evidenced by a more natural countenance, quiet sleep, and a return of sensibility. On the other hand, we may apprehend a fatal termination when the stools continue frequent and unnatural, the vomiting incessant, the pulse small, thready and irritated, and especially where the brain exhibits its participation in the diseased action by delirium, coma, insensibility, or convulsions. Dr. Dewees has pointed out "a symptom, which, as far as he has observed, has always proved fatal: it is a crystalline eruption upon the chest, of an immensity of watery vesicles, of a very minute size." "Another symptom which is much more common but not less fatal," says Dr. Dewees, "is the thrusting of the fingers into the back part of the mouth as if desirous of removing something from the throat: and he mentions a third, which is the escape of a living worm, in the chronic stage of this affection. If the worm comes away dead," says he, "there is nothing in the circumstance; but if alive, it is a deadly sign."

In attempting an elucidation of the *pathology* of cholera infantum, several very important facts demand a due consideration: And first I may remark, that the disease invariably occurs during the warm weather of our summer months—hence its vulgar title "the summer complaint." Commencing in the month of June or July, and when the thermometer is at its highest range, it continues with increasing violence until the appearance of frost. "A cool day," says Dr. Rush, "frequently abates its violence, and disposes it to a favourable termination." Secondly, this disease always attacks children during primary dentition. This fact is conceded on all hands, but it is not, in my opinion, invested with the importance it deserves. It has been remarked by all writers on this disease, and I may fearlessly appeal to the experience of all present for its confirmation, that previous to the commencement of the process of dentition, and after its completion, cholera infantum very seldom makes its attack. Thirdly, I would remark, that this affection very generally prevails in our large cities, and usually in its most confined courts and alleys and in houses badly ventilated and filthy. In confirmation of this fact, I have only to quote the language of Dr. Eberle. "During a practice of twelve years in the country," he says, "I met with but two or three cases of this disease." And all admit, that the only sure remedy, in the management of cholera infantum, consists in a removal from the contaminated air of our crowded cities, into the pure and free air of the country. We have, then, three remote causes, whose united influence is absolutely necessary to the production of this malady, namely, high atmospheric temperature, the irritation produced by dentition, and the impure air of crowded cities. Any one or even two of these causes, without the aid of the third, are insufficient to the production of the disease: and although cases do occasionally occur, under circumstances favouring an opposite construction, yet they are too rare to form an exception to the general rule—or we might say with the ancient Romans, "exceptio probat regulam." A slight review of the effects of these prolific sources of disease will, perhaps, enable us to arrive at a satisfactory explanation of the pathology of this affection.

First, high atmospheric temperature excites the capillaries to increased secretion. This inordinate excitement produces a copious flow of perspiration, which is extremely liable, from exposure to cool or damp night air, to receive a sudden check. Heat, too, is known to stimulate the liver to increased excitement, and a superabundance of bile is poured into the intestines. "When the cutaneous and hepatic functions, while in a state of inordinate activity, are suddenly arrested by the influence of cold, the blood retreats from the surface to the internal vessels: the portal circulation becomes engorged, and the capillaries of the mucous membrane of the bowels strongly congested. This injected or engorged state of the capillaries of the mucous membrane of the alimentary canal gives rise, we may presume, to morbid irritability of this structure, and consequently the characteristic phenomena of the disease." Heat may also be said to render the whole system languid and irritable, and in a situation, favourable to the operation of the exciting causes of disease.

Secondly, the effect of dentition, in almost every infant, is to a greater or less degree, irritation. This irritation may affect the brain, producing eruptions about the ears, delirium, or convulsions: or it may operate upon the stomach and bowels, causing, as we very frequently observe, vomiting and a diarrhœa. Should the bowels continue undisturbed during dentition, a proportionably severe irritation of the brain is to be apprehended, and we endeavour to fulfil the indications of nature by the interposition of purgatives, and thus divert action from the brain to the alimentary canal.

Of the operation of the impure air of crowded cities in the production of this disease, we must profess our ignorance. Of the fact, however, there can be no doubt. It most probably acts, by rendering the infantile system irritable and peculiarly predisposed to suffer from the irritation of dentition.

"We may therefore," in the language of an excellent writer upon this disease, "presume that in the irritable condition of the system, produced by a very warm and contaminated atmosphere, dentition causes more or less cerebral irritation, which being reflected on the stomach and bowels, renders them preternaturally irritable. If in this state of the alimentary canal, the cutaneous exhalants are over-excited and debilitated by high atmospheric temperature, the slightest reduction of temperature, a current of fresh air, or damp night air, will readily cause a sudden torpor of these emunctories. The blood will retreat from the surface to the internal organs, and give rise to engorgement of the vessels of the liver and mucous membrane of the bowels, by which the gastro-intestinal irritability will be still further increased, and the characteristic symptoms of the disease excited."

In infants, predisposed to this disease, an attack may be readily induced, by improper exposures to cool or damp night air, by light clothing, improper and indigestible food, worms.

*Dissections* plainly reveal the nature of this disease. The brain is often found, in cases of long standing, congested, and in some instances, there are discovered effusions of water in the ventricles. In recent cases, however, the brain is but slightly affected. It is upon the liver and alimentary canal that the strongest marks of disease are apparent. The mucous membrane of the stomach and bowels is almost uniformly inflamed and congested. The liver is found engorged with blood and much increased in size. The gall bladder is usually filled with bile, which may be tenacious and of a dark green colour, or limpid and colourless.

*Treatment.* The leading indications in the treatment of this disease, appear to be: 1st. To destroy the influence of the remote causes; 2d. To allay the cerebral irritation; 3d. To calm the irritability of the stomach and bowels; 4th. To restore the action of the liver; and, 5th. To give tone to the stomach and bowels.

It is obvious, that so long as the patient is exposed to the combined influence of heat, dentition, and impure air, complete recovery cannot be reasonably expected. Our first object, then, should be to remove, or to palliate these causes. This may be most effectually accomplished, first, by removal to the country. Pure country air is, unquestionably, the most effectual remedy we possess for the relief of this disease, and will alone, in the majority of cases, effect a permanent cure. Dr. Rush says, that " out of the many hundred children whom I have sent into the country, in every stage of this disease, I have lost but three. But as it is not always in the power of our patients to command the advantages of a country residence, we should direct that the child be carried into the fresh air two or three times every day, either in the arms of its nurse, or in an open vehicle, or in a boat. Dr. Chapman speaks highly of the advantages to be derived from sailing, and states that he has known a great number of cases effectually cured by sailing backwards and forwards to Camden. The patient too, should be placed in the largest and best ventilated room in the house, and the windows and doors constantly kept open during the day. The damp night air, however, should be carefully excluded. Cleanliness should also be strictly observed, both in the patients person, and in his clothing, which should be regulated by the weather. Having tated that the irritation of dentition is deeply concerned in the production of this disease, I need scarcely say that the gums should be frequently and carefully examined, and if inflamed or swelled, they should be freely lanced. These remedies will sometimes immediately arrest the progress of the disease, and render all further interference unnecessary. But should the disease become established, and the system sympathise in the morbid action, medicinal treatment will become absolutely necessary. To allay the cerebral irritation, without which it is impossible to calm the irritability of the stomach and bowels, is the first object to which our remedies should be directed. For this purpose, should fever supervene, the cheeks become flushed, the eyes fierce and the head hot, venesection will be necessary. Bleeding from the arm, however, is rarely demanded, and should never be carried to a great extent. Much more advantage may be obtained from the employment of leeches to the temples or nape of the neck or abdomen, and followed by the application of blisters behind the ears. These remedies will frequently allay the cerebral irritation, and render the disease much more mild and manageable in its course. Dr. Eberle lays great stress upon the employment of these remedies, and I am persuaded from my own experience with great reason.

It was formerly very much the practice to commence the treatment of this disease, with an emetic, but I am happy to find these remedies falling into general disuse in the management of the disease. Their employment was predicated upon an erroneous view of the pathology of this affection, and, what is still more to the purpose, they were found to be productive of effects, manifestly injurious. Where, however, the stomach is loaded with foul matter, and the efforts of nature are insufficient to its rejection, the vomiting may be promoted by the free exhibition of diluents. Our great object, however, should be to arrest the vomiting as speedily as possible, in order to allow the administration of other remedies. This object will often be successfully accomplished, by the application of sinapisms, spice plasters, emollient poultices, or fomentations of hops to the abdomen. Should these fail, however, the usual anti emetics may be administered, among which may be mentioned lime water and milk, strong coffee, the essence or infusion of mint. Dr. Condie, in a very excellent paper upon this disease, read before this society, and afterwards published in the American Journal of Medical Sciences, very strongly recommends the spirits of turpentine, in doses of from ten to thirty drops, repeated three or four times in the day, and my own experience bears testimony to its utility. The remedy upon which Dr. Dewees mainly depends for the relief of the vomiting, is an enema of a gill of warm water, holding in solution three teaspoonsful of common salt. This I have never tried, but from the extensive experience of Dr. Dewees, would not hesitate in receiving it as a valuable remedy. Having succeeded by these means in tranquilising the stomach, our next object is to restore the healthy action of the liver. Of all the remedies we possess for this purpose, none can compare with calomel. The smallness of the dose requisite to produce its effects, its weight, and its want of taste, render it liable to be retained in the stomach, when every thing else would be rejected. These are not however the only advantages it possesses over all other cathartics. It exerts a specific and powerful action upon the liver, disgorges its vessels, promotes the secretion of bile, corrects the vitiated secretion of the intestinal capillaries, and thus attacks the disease in its very citadel. To obtain the full effects of calomel, it should be administered in minute doses, and repeated as often as the urgency of the symptoms may require: from one eighth to a quarter of a grain repeated every half hour or hour, has, in my hands, displayed extraordinary powers. Much as is to be obtained from the exhibition of calomel alone, its powers are increased by combination with ipecacuanha. The good effects of ipecacuanha in relieving intestinal disorder, in correcting vitiated secretions, and in tranquillizing any undue irritation or excitement, are well known. In dysentery, diarrhœa, and cholera morbus, it is often productive of the happiest effects, and in the disease under consideration it displays powers no less efficacious. I have generally been in the habit of using the following prescription. R. Hydr. Subm. grs. iij. ipecac.

pulv. grs. iv to vi.—Pulv. Gum Arab. grs. xv.—m. in chart. no. xij. divid. Of these, I usually direct one to be taken every half hour or hour, according to the violence of the symptoms. The use of this prescription should be steadily persevered in until the discharges from the bowels present the appearance of bile. As soon as this takes place, there is a manifest amelioration of the symptoms. The expression of the countenance becomes natural, the skin moist, and of an uniform temperature, the cerebral irritation subsides and the frequency and pain of the intestinal evacuations will be diminished. The following prescription has been highly extolled by many practitioners in this city, and I believe with just cause. R. Calomel gr. j. Ipecac. pulv. gr. j. a ij. Sacch. Saturni grs. xij. In chart. no xij. divid. Dose, one every hour or two according to the urgency of the symptoms. It is particularly efficacious in the dysenteric form. Should the disease, however, have made its attack suddenly, and with severity, exhibiting symptoms of cerebral concussion and there be reason to apprehend a loaded state of the bowels, it will be proper to commence, without loss of time, with large and repeated doses of calomel, with a view to its purgative operation. Let bile be once observed in the stools, and we have no fear for the result of the case: and until bilious discharges are procured, it matters not how mild the symptoms may appear, there is a secret enemy lurking in the system, which will sooner or later destroy its victim, at the very moment, perhaps, when we are fondly anticipating a complete recovery. The evacuations, from the bowels, then, should be frequently and carefully examined, and until the stools become decidedly billious, the use of calomel should be steadily persisted in. As soon as this change is perceived in the stools, a dose of olive or castor oil should be administered, and repeated as often as the state of the bowels requires it. The spiced syrup of rhubarb will often be found an useful remedy in this stage of the disease, and will be readily taken when, perhaps, almost every other medicine creates nausea. Having, by these means, procured the natural and healthy action of the chylopoietic viscera, it has been very much the custom with practitioners to resort to opiates and astringents. These remedies, however, I would sedulously avoid, being persuaded that the mortality of this disease is principally to be ascribed to their improper employment. From the liability of the brain to take in diseased action, it is obvious, that these remedies cannot fail in the early stages to do harm, by determining action to the brain, and thus causing the very symptoms we are anxious to allay. The use of opium, I know, has the sanction of high names, but from my own experience I should say, that we cannot be too cautious in its administration. When the cerebral irritation, together with the arterial action, is entirely removed, and the disease runs into what Dr. Chapman denominates a gleet of the bowels, then indeed, opiates and astringents may be advantageously resorted to. But in the earlier stages of this affection, they are altogether inadmissible. Judging from experience, I should say, that at this period, anodyne or bland enemata are decidedly the best means we can employ to check the inordinate action of the intestines. Enemata of melted butter, or of starch, or flaxseed tea, with a few drops of laudanum, are frequently of great advantage. The warm bath has been highly recommended in every stage of this disease, and I am confident that I have derived great benefit from its employment. Its efficacy appears to depend upon its unloading the vessels of the abdominal viscera, determining to the skin and equalizing the circulation. Drs. Rush and Miller have proposed the use of the cold bath or sponging the surface with cold water, but I have never had occasion to test its virtues. In cases attended with undue determination of blood to the brain, I have frequently, and I think with advantage, applied cold to the head. At the same time, in order more effectually to divert the action from the brain, the application of sinapisms or blisters to the abdomen or extremities, often proves of signal benefit. To restore tone to the stomach in the sinking stages of the disease, mild tonics and stimulants will become necessary. For this purpose, the infusion of bark, columbo, gentian or chamomile with the addition of some aromatic, may be administered. Among the astringents may be mentioned as most proper in the latter stages of this affection, decoctions or infusions of dewberry root, wild cherry tree bark, logwood, and in the lowest stages, kino and galls. The effects of these tonic and astringent remedies should be seduously watched, and if fever or cerebral affection supervene, they should be immediately withheld. Dr. Condie speaks highly of the effects of charcoal in this stage of the disease. "Under the employment of this remedy," says he, "the stools soon become more natural in appearance, and diminished in frequency, while the appetite for food, and the digestive powers of the stomach, are sensibly increased." I have never yet made trial of this remedy in cholera infantum, but have no doubt of its utility, when properly administered. It is said to be most advantageously prescribed in combination with rhubarb and ipecacuanha.

To relieve flatulence of the stomach and bowels, which is a very distressing symptom, recourse may be had to spirits of turpentine, soot tea, oil of Juniper, cinnamon or mint-water, small doses of volatile alkali, or a small quantity of sound sherry or Madeira wine, or of pure anise-seed cordial. After all, however, it is upon a properly regulated diet, that our chief dependence is to be placed, in the latter stages of this affection. Without unremitted attention to the diet of the patient, all our remedies will prove inefficacious, and our efforts at relief be in vain. If the child has not been weaned, it should be confined entirety to the breast of the mother or nurse. The generality of cases of this disease which come under our notice, however, occur after the child has been deprived of his maternal nutriment: this being the case, the most bland and digestible articles of food should be selected. Boiled milk alone, or with the addition of a little flour and loaf sugar, arrow root, tapioca, sago, and fresh whey constitute wholesome and salutary articles of diet. The addition of wine and spices contribute in rendering these several articles more palatable, and may prove useful by their astringent effects upon the bowels. Beef-tea and chicken-water are in some instances very agreeable, and may be allowed without injury. "We often see," says Dr. Rush, "an appetite suddenly awakened for articles of diet of a stimulating nature." He states that he has seen many children recover, from being gratified in an inclination to eat salted fish, and the different kinds of salted meat. "In some instances," he says, "they discover an appetite for butter, and the richest gravies of roasted meats, and eat them with obvious relief to all their symptoms: and he cites the case of a child, who was perfectly restored from the lowest stage of the disease, by eating large quantities of rancid English cheese, and drinking two or three glasses of port wine every day. The child would in no instance eat bread with the cheese, nor taste the wine if it was mixed with water. This craving of the stomach appears to be an indication of nature, which should, in chronic cases, by no means be disregarded.

The means of prevention of an attack of cholera infantum consist, 1st, in the proper regulation of the diet. If the health and condition of the mother will admit of it, the child should be confined to the breast until the process of dentition is nearly completed. Having been weaned, however, the diet should be simple and nutritive. 2d. During dentition, the gums should be frequently and carefully examined, and if they should appear swollen or inflamed, they should be freely lanced. The child should be exposed every day to the fresh air, by taking it into the country or by crossing the river. Dr. Rush says, that out of the many hundred children, that he has sent to the country to avoid an attack of this disease, he has never known an instance in which it occurred. 4th. The clothing shonld be propealy regulated and adapted to the vicissitudes of the weather. Many children, I am inclined to believe, have escaped an attack of cholera infantum by wearing flannel next to the skin. Dr. Chapman urges the use of a flannel roller applied around the hips and abdomen. 5th. Perhaps the most important preventive of this disease consists in the daily use of the cold bath. It promotes the healthy action of the skin, invigorates the stomach and bowels, produces cleanliness, and gives tone to the whole system.

